# Brain-derived neurotrophic factor expression predicts adverse pathological & clinical outcomes in human breast cancer

**DOI:** 10.1186/1475-2867-11-23

**Published:** 2011-07-18

**Authors:** Neill Patani, Wen G Jiang, Kefah Mokbel

**Affiliations:** 1 Department of Breast Surgery, The London Breast Institute, The Princess Grace Hospital, 42-52 Nottingham Place, W1U-5NY, London, England, UK; 2 Metastasis & Angiogenesis Research Group, University Department of Surgery, Cardiff University School of Medicine, Cardiff University, Heath Park, CF14-4XN, Cardiff, Wales, UK; 3 Department of Biosciences, School of Health Sciences and Social Care, Brunel Institute of Cancer Genetics and Pharmacogenomics, Brunel University, Uxbridge, Middlesex, UB8 3PH, London, England, UK

## Abstract

**Introduction:**

Brain-derived neurotrophic factor (BDNF) has established physiological roles in the development and function of the vertebrate nervous system. BDNF has also been implicated in several human malignancies, including breast cancer (BC). However, the precise biological role of BDNF and its utility as a novel biomarker have yet to be determined. The objective of this study was to determine the mRNA and protein expression of BDNF in a cohort of women with BC. Expression levels were compared with normal background tissues and evaluated against established pathological parameters and clinical outcome over a 10 year follow-up period.

**Methods:**

BC tissues (n = 127) and normal tissues (n = 33) underwent RNA extraction and reverse transcription, BDNF transcript levels were determined using real-time quantitative PCR. BDNF protein expression in mammary tissues was assessed with standard immuno-histochemical methodology. Expression levels were analyzed against tumour size, grade, nodal involvement, TNM stage, Nottingham Prognostic Index (NPI) and clinical outcome over a 10 year follow-up period.

**Results:**

Immuno-histochemical staining revealed substantially greater BDNF expression within neoplastic cells, compared to normal mammary epithelial cells. Significantly higher mRNA transcript levels were found in the BC specimens compared to background tissues (p = 0.007). The expression of BDNF mRNA was demonstrated to increase with increasing NPI; NPI-1 vs. NPI-2 (p = 0.009). Increased BDNF transcript levels were found to be significantly associated with nodal positivity (p = 0.047). Compared to patients who remained disease free, higher BDNF expression was significantly associated with local recurrence (LR) (p = 0.0014), death from BC (p = 0.018) and poor prognosis overall (p = 0.013). After a median follow up of 10 years, higher BDNF expression levels were significantly associated with reduced overall survival (OS) (106 vs. 136 months, p = 0.006). BDNF emerged as an independent prognostic variable in multivariate analysis for disease free survival (DFS) (p = 0.026) and approached significance for OS (p = 0.055).

**Conclusion:**

BDNF expression was found to be significantly higher in BC specimens compared to normal tissue. Higher transcript levels were significantly associated with unfavourable pathological parameters including nodal positivity and increasing NPI; and adverse clinical outcomes including LR, death from BC, poor prognosis, reduced DFS and OS. BDNF offers utility as a prognostic marker and potential for targeted therapeutic strategies.

## Introduction & Background

Brain-derived neurotrophic factor (BDNF) belongs to the neurotrophin (NT) superfamily of polypeptide growth factors, which includes nerve growth factor (NGF) and NTs 3-6 [1,2]. NTs and their receptors have key physiological roles in the development and function of the central and peripheral nervous systems in vertebrates [3-5]. However, they are also widely expressed in non-neuronal tissues [6]. Expression of the BDNF gene (*BDNF*) is regulated by the presence of multiple activity dependent and tissue-specific promoters [7]. BDNF signals preferentially via its high affinity tyrosine kinase receptor, tropomyosin receptor kinase B (TrkB). Ligand-induced receptor dimerisation results in auto-phosphorylation and initiates multiple signalling cascades, including the mitogen-activated protein kinase (MAPK), phosphatidyl-inositide 3-kinase (PI3K) and phospholipase C-gamma (PLC- γ) pathways, that promote cellular survival [8-11]. However, BDNF also shares a common low affinity receptor (p75^NTR^) with the other NTs, which is a member of the tumor necrosis factor (TNF) receptor superfamily, implicated in the modulation of cell survival, cell cycle regulation and cytoskeletal rearrangement [8]. The overall cellular response to BDNF exposure is therefore likely to reflect an equilibrium between TrkB and p75^NTR ^activity.

BDNF has been implicated in various human pathologies, including: depression, epilepsy, Alzheimer's, Parkinson's and Huntington's disease [7,12]. BDNF has also been associated with several human cancers, both neuronal and non-neuronal, including: neuroblastoma [13], myeloma [14], ovarian [15,16], lung [17], prostate [18], hepato-cellular [19], pancreatic [20][21,22], head and neck squamous cell carcinomas [23] and pulmonary carcinoid tumours [24]. Interestingly, the archetypal neurotrophic factor NGF has been demonstrated to stimulate proliferation, angiogenesis and behave as an anti-apoptotic factor in human breast cancer (BC) [1,25-27] with potential for therapeutic targeting [28]. In keeping with this, BDNF has been associated with cell survival in human BC cell lines [1]. BDNF has also been demonstrated to be significantly up regulated in oestrogen receptor alpha (ER-α) positive BCs [29]. Although increased NT and cognate receptor expression have been demonstrated in BC [2,30], the precise biological role of BDNF and its utility as a novel biomarker have yet to be determined. The objective of this study was to determine the mRNA and protein expression of BDNF in a cohort of women with BC. Expression levels were compared with normal background tissues and evaluated against established pathological parameters and clinical outcome over a 10 year follow-up period.

## Methods

### Patients

BC tissues (n = 127) and normal background tissues (n = 31) were collected from University Hospital of Wales and St George's Hospital and Medical School; institutional guidelines, including ethical approval and informed consent, were followed. Specimens were obtained immediately after excision during surgery and stored at -80°C until use. A consultant pathologist examined haematoxylin and eosin stained frozen sections to verify the presence of tumour cells in the collected samples. Normal tissue was derived from the background breast parenchyma of BC patients within the study group. Medical notes and histology reports were used to extract the clinico-pathological data (Table 1). A customized database was established to record the data.

**Table 1:** Clinical and pathological data

Parameter	Category	Number
**Node Status**	Node positive	54
	Node negative	73

**Tumour Grade**	1	24
	2	43
	3	58

**Tumour Type**	Ductal	98
	Lobular	14
	Medullary	2
	Tubular	2
	Mucinous	4
	Non specific	7

**TNM staging**	1	70
	2	40
	3	7
	4	4

**NPI**	NPI1	68
	NPI2	38
	NPI3	16

**Clinical Outcome**	Disease free	90
	Alive with metastasis	7
	With local recurrence	5
	Died from breast cancer	16
	Died of unrelated disease	9

**ER status**	ER α negative	75
	ER α positive	38
	ER ß negative	91
	ER ß positive	24

Note: missing values reflect discarded/un-interpretable values

### Tissue Processing, RNA Extraction, cDNA Synthesis & RT-PCR

Frozen sections of tissue were cut at a thickness of 5-10 mm and kept for routine histological analysis. Additional 15-20 sections were mixed and homogenized using a hand-held homogenizer in ice-cold RNA extraction solution. RNA from cells was extracted using an RNA extraction kit (AbGene Ltd, Surrey, England, UK). RNA concentration was quantified using a UV spectrophotometer (Wolf Laboratories, York, England, UK). Reverse transcription was carried out using a reverse transcription kit, cDNA was synthesised using first strand synthesis with an anchored oligo^dt ^primer (AbGene, Surrey, UK). The polymerase chain reaction (PCR) was performed using sets of primers (Table 2) with the following conditions: 5 min at 95°C, 20 seconds at 94°C, 25 seconds at 56°C, 50 seconds at 72°C for 36 cycles and finally 72°C for 7 minutes. ß-actin was amplified and used as a house keeping control to verify the quality of cDNA. PCR products were separated on a 0.8% agarose gel, visualised under UV light, photographed using a Unisave™ camera (Wolf Laboratories, York, England, UK) and recorded with Photoshop software.

**Table 2:** Forward and reverse primers

BDNF F	ACATCATTGGCTGACACTTT
BDNF Zr	ACTGAACCTGACCGTACATGCGTCCTTATTGTTTTCTT

CK-19 F	CAGGTCCTAGAGGTTACTGAC

CK-19 Zr	ACTGAACCTGACCGTACACACTTTCTGCCAGTGTGTCTTC

β-actin F	ATGATATCGCCGCGCTCGTC

β-actin Zr	CGCTCGGTGAGGATCTTCA

### Quantitative Analysis of BDNF

BDNF transcript levels within the above-prepared cDNA were determined using real-time quantitative PCR, based on the Amplifluor™ technology, modified from previous reports [31,32]. Pairs of PCR primers were designed using the Beacon Designer™ software (Version 2, Palo Alto, California, USA) and synthesized by Sigma-Aldrich, added to the reverse primer was an additional sequence, known as the Z sequence (5'-ACTGAACCTGACCGTACA-'3) which is complementary to the universal Z probe (Intergen Inc., Oxford, England, UK). The product expands one intron (Table 2). Taqman detection kit for ß-actin was purchased from Perkin-Elmer. The reaction was carried out using the following: custom made hot-start Q-master mix Abgene (Surrey, England, UK), 10 pmol of specific forward primer, 1 pmol reverse primer with the Z sequence (Table 2), 10 pmol of FAM- (fluorogenic reporter dye, carboxyfluorescein) tagged probe (Intergen Inc.), and cDNA generated from 50 ng RNA. The reaction was carried out using IcyclerIQ™ (Bio-Rad, Hemel Hempstead, England, UK) which is equipped with an optical unit that allows real-time detection of 96 reactions, under the following conditions: 94°C for 12 minutes, 50 cycles of 94°C for 15 seconds, 55°C for 40 seconds and 72°C for 20 seconds. The transcript levels were generated from an internal standard that was simultaneously amplified with the samples. The levels of gene expression were then normalized against the housekeeping control CK-19, which was also quantified in these specimens, to correct for varying amounts of epithelial tissue between samples [33]. With every PCR run, a negative control without a template and a known cDNA reference sample as a positive control, were included.

### Immuno-histochemical Analysis of BDNF in Tissues

Frozen sections of breast tissues (normal and tumour) were cut at a thickness of 6µm using a cryostat. Sections were mounted on super frost plus microscope slides, air dried and then fixed in a mixture of 50% Acetone and 50% methanol. Sections were then placed in Optimax wash buffer (Biogenex) for 5-10 minutes to rehydrate. Sections were incubated for 20 minutes in a 10% horse serum blocking solution and then probed with the primary antibody, anti-human BDNF (H-117, SC-20981, Santa Cruz Biotechnologies, Inc.), dilution 1:150 in Optimax buffer (Biogenex). Sections were rinsed thoroughly with wash buffer, before incubation for 30 minutes in the secondary biotinylated antibody (Multi-link Swine anti-goat/mouse/rabbit immunoglobulin, Dako Inc.). Following thorough washings, sections were incubated for 30 minutes with Avidin Biotin Complex (Vector Laboratories) followed by further washings. Di-amino-benzidine (DAB) chromogen (Vector Labs) was then added to the sections which were incubated in the dark for 5 minutes. Sections were then washed for 10 minutes in running tap water, prior to nuclear counter staining with Gill's Haematoxylin for 1 minute, followed by a further 10 minute wash with tap water. Sections were dehydrated in ascending grades of methanol before clearing in xylene and mounting under a cover slip using clear mounting medium.

### Statistical Analysis

The two-sample *t*-test (comparison of mean copy number) was used for statistical analysis of absolute and normalised gene copy number. For normality the Anderson-Darling test was used. The transcript levels within the BC specimens were compared to normal background tissues and analyzed against conventional pathological parameters and clinical outcome over a 10 year follow-up period. In each case the true copy number was used for statistical analysis and hence the samples were not classified as positive or negative. The statistical analysis was carried out using Minitab version 14.1 (Minitab Ltd. Coventry, England, U.K.) using a custom written macro (Stat 2005.mtw). For purposes of the Kaplan-Meier survival analysis, the samples were divided arbitrarily into two groups, 'high transcript level' or 'low transcript level'. The cut-off was guided by the Nottingham Prognostic Index (NPI) value with which the value of the moderate prognostic group was used as the dividing line at the start of the test. NPI = tumour size (cm) × 0.2 + lymph node stage (1 - no nodes affected; 2 - up to 3 nodes affected; 3 - more than 3 nodes affected) + Grade (1-3, Scarff-Bloom-Richardson). NPI scores were classified into three groups: <3.4 = NPI-1, 3.4-5.4 = NPI-2, >5.4 = NPI-3. Survival analysis was performed using SPSS version 16.0 (SPSS Inc. Chicago, IL, USA).

## Results

**Figure 1:**
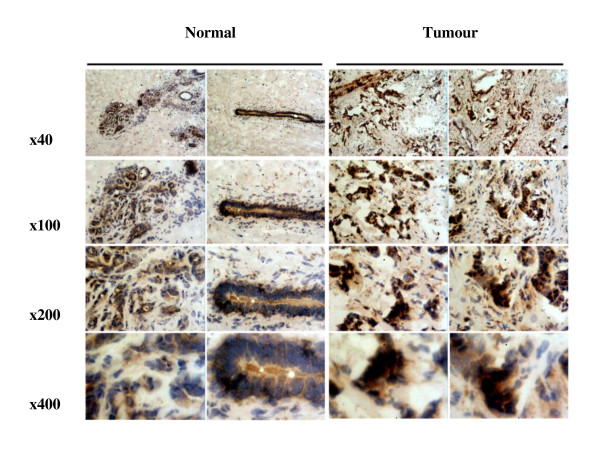
**Immuno-histochemical analysis of BDNF expression and localization in BC and normal mammary tissues**.

**Figure 2:**
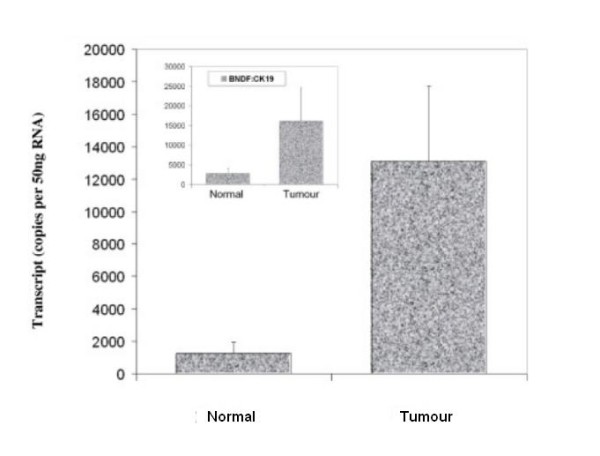
**Summary of BDNF expression profiles for normal and tumour specimens**. Values represent the true copy number of mRNA transcripts, absolute and normalised against CK-19 (inset), expressed as mean and standard deviation.

**Figure 3:**
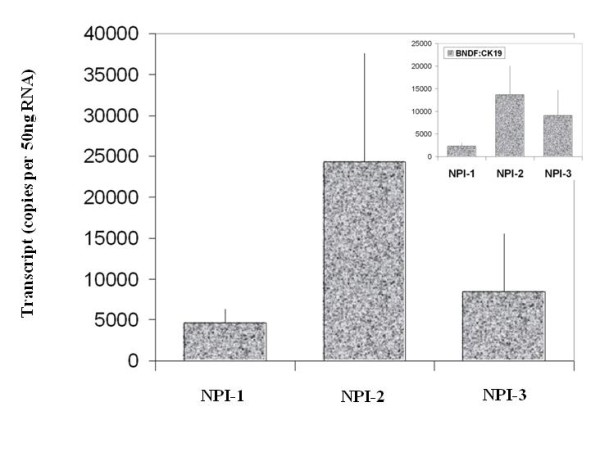
**Summary of BDNF expression profiles and NPI**. Values represent the true copy number of mRNA transcripts, absolute and normalised against CK-19 (inset), expressed as mean and standard deviation.

**Figure 4:**
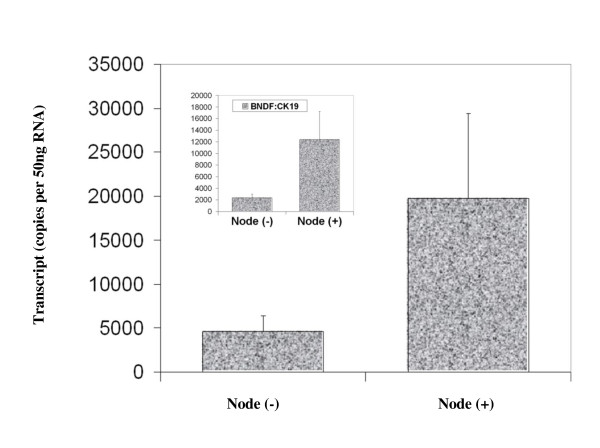
**Summary of BDNF expression profiles and nodal status**. Values represent the true copy number of mRNA transcripts, absolute and normalised against CK-19 (inset), expressed as mean and standard deviation.

**Figure 5:**
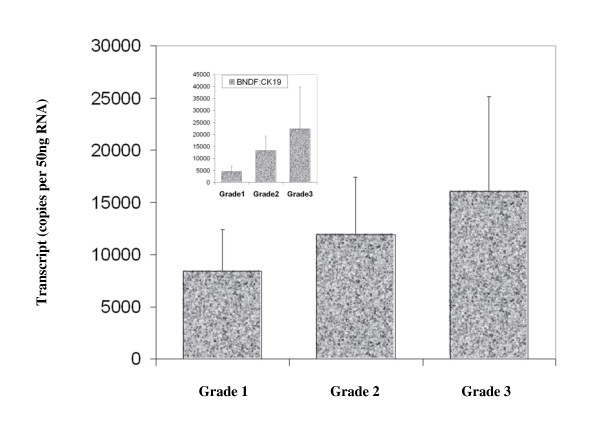
**Summary of BDNF expression profiles and tumour grade**. Values represent the true copy number of mRNA transcripts, absolute and normalised against CK-19 (inset), expressed as mean and standard deviation.

**Figure 6:**
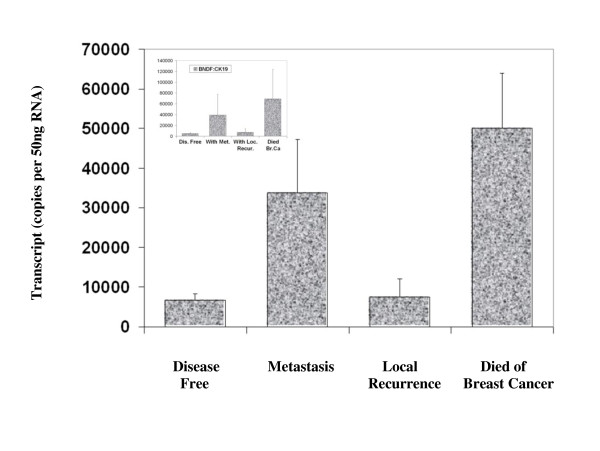
**Summary of BDNF expression profiles and clinical outcomes**. Values represent the true copy number of mRNA transcripts, absolute and normalised against CK-19 (inset), expressed as mean and standard deviation.

**Figure 7:**
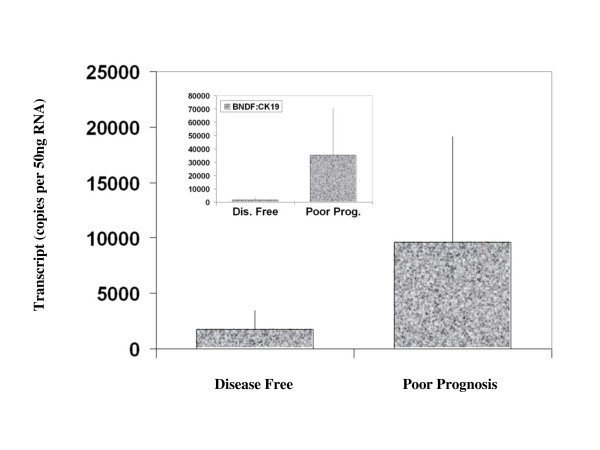
**Summary of BDNF expression profiles and poor prognosis (LR, metastases or death from BC)**. Values represent the true copy number of mRNA transcripts, absolute and normalised against CK-19 (inset), expressed as mean and standard deviation.

BDNF was found to be expressed in both normal breast tissue and BC specimens. Immunohistochemical staining for BDNF was substantially more positive within the neoplastic cells of breast tissues than in normal mammary epithelial cells (Figure 1). BDNF mRNA transcript levels were determined both in absolute terms and normalised against CK-19 in order to correct for varying amounts of epithelial tissue between samples. Significantly higher mRNA transcript levels were found in the BC specimens compared to the background tissue (absolute mean copy number 13104 vs. 1262, p = 0.007) (Figure 2). The expression of BDNF mRNA was demonstrated to increase with increasing NPI; NPI-1 vs. NPI-2 (normalised mean copy number 2339 vs. 13690, p = 0.009) and approached significance for NPI-1 vs. NPI-3 (absolute mean copy number 4586 vs. 8442, p = 0.065) (Figure 3). Increased BDNF transcript levels were found to be significantly associated with nodal positivity (normalised mean copy number 2339 vs. 12378, p = 0.047) (Figure 4). Although both absolute and normalised BDNF transcript levels were found to increase with tumour grade, this did not reach statistical significance (Figure 5). Compared to patients who remained disease free, higher BDNF expression was significantly associated with local recurrence (LR) (absolute mean copy number 6660 vs. 7430, p = 0.0014), death from BC (absolute mean copy number 6660 vs. 49945, p = 0.018) and approached significance for those developing metastases (absolute mean copy number 6660 vs. 33787, p = 0.078) (Figure 6). Overall, significantly higher transcript levels were found in patients with poor prognosis (LR, metastases or death from BC) compared to those who remained disease free (absolute mean copy number 6660 vs. 37243, p = 0.013) (Figure 7). The expression of BDNF was not found to increase significantly with increasing TNM stage.

**Figure 8:**
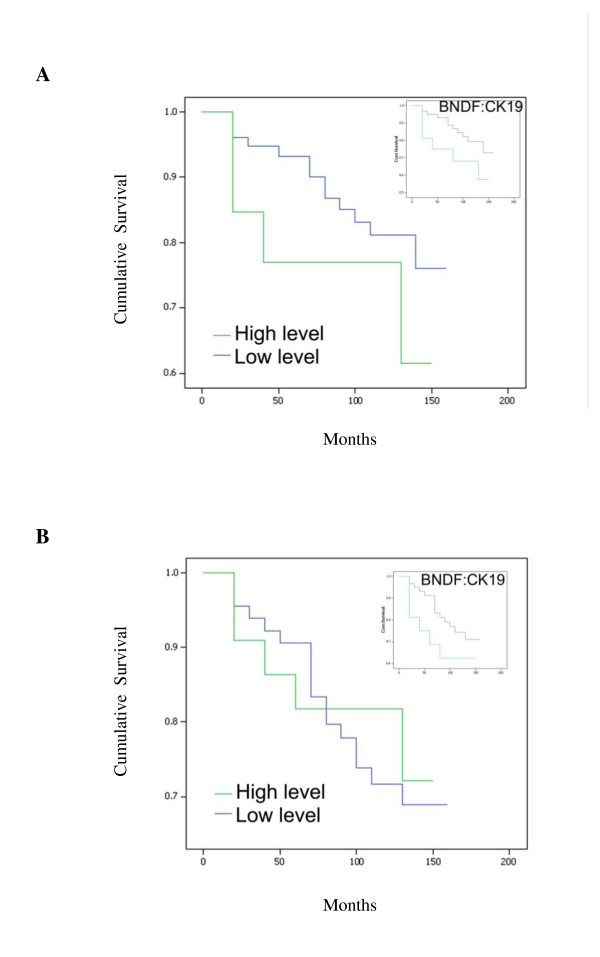
**BDNF expression levels and Kaplan-Meier analysis for OS (A) and DFS (B), absolute and normalised against CK-19 (inset)**.

After a median follow up of 10 years, higher BDNF expression levels, both absolute and normalised, were significantly associated with reduced overall survival (OS) times: higher absolute expression levels, mean survival = 125.36 (95% CI = 107.49-143.24) vs. 134.31 (95% CI = 123.52-145.11) months, p = 0.041; higher normalised expression levels, mean survival = 105.55 (95% CI = 78.88-132.21) vs. 135.97 (95% CI = 125.07-146.87) months, p = 0.006 (Figure 8A). The disease free survival (DFS) curves for women with tumours which were classified as having 'high levels' of BDNF transcript was not found to differ significantly from that of their 'low level' counterparts. The survival curves show higher levels of BDNF were of marginal benefit in predicting lower DFS: higher normalised expression levels, mean survival = 102.13 (95% CI = 74.76-129.49) vs. 130.67 (95% CI = 118.79-142.56) months, p = 0.137, NS (Figure 8B). The independent prognostic utility of BDNF in multivariate analysis was statistically significant for DFS (p = 0.026) and narrowly fell short of significance for OS (p = 0.055) (Table 3).

**Table 3:** Multivariate analysis of prognostic parameters

Parameter	Overall Survival (p value)	Disease Free Survival (p value)
BDNF status	0.055	0.026

NPI	0.093	0.035

ER status	0.57	0.042

Grade	0.85	0.079

Stage	0.12	0.308

## Discussion

NTs and their receptors are increasingly being implicated as novel mediators of carcinogenesis in neuronal and non-neuronal tissues. Whilst the literature regarding BC remains sparse, altered expression and function of these factors are likely to contribute to tumourigenesis and progression. The present study adds to the literature in support of the oncogenic function of BDNF in BC. Furthermore, this study is the first to quantitatively evaluate BDNF mRNA expression in a large cohort of BC patients and provide correlation with conventional pathological parameters and clinical outcomes over an extended follow-up period. Significantly higher mRNA transcript levels were found in BC specimens compared to normal tissue, corroborated at the protein level by immuno-histochemical staining. BDNF expression was found to increase with increasing NPI, nodal positivity, LR, death from BC and poor prognosis overall. After a median follow up of 10 years, higher BDNF expression levels were significantly associated with reduced OS. Furthermore, BDNF remained an independent prognostic variable in multivariate analysis. Our results differ from those of Blasco-Gutierrez et al. [2] who did not identify differential staining between tumour and normal breast tissue and reported no association between BDNF expression and pathological parameters or clinical outcomes. In their study, Tozlu et al. [29] employed real-time quantitative reverse transcription (RT)-PCR to compare the mRNA expression of 560 selected genes in BCs excised from 48 women. BDNF emerged as a growth factor significantly up regulated amongst ER-α positive tumours in both the screening and validation sets. However, the relationship was found to be reversed *in vitro*, where BDNF was found to be up regulated in ER-α negative BC cell lines. Interestingly cross-talk between steroid and growth factor pathways has been identified in the brain, with oestrogen implicated in the regulation of BDNF and demonstrating some degree of overlap in activity with NTs [34]. BDNF has also been associated with increased cell survival, although not proliferation, in human BC cell lines. In accordance with the fact that BDNF did not demonstrate a mitogenic effect, no expression of TrkB was found in any of the BC cells tested. However, the interaction between BDNF and P75^NTR^, which specifically induces NF-ϰB, has been implicated in the protection of BC cells from apoptosis [1]. Further support for the oncogenic function of BDNF *in vivo *comes from a transgenic mouse model of spontaneous mammary tumor formation, following exposure to the organochlorine pesticide Dieldrin, which is a persistent environmental toxin thought to increase the risk of BC [35]. Offspring of these mice show enhanced development of mammary tumours with increased mRNA and protein expression of BDNF and TrkB [36].

The factors regulating NT and NT receptor expression in normal and malignant breast tissues remain unknown. Whilst the precise biological role of the BDNF/TrkB/P75^NTR ^axis in human BC has yet to be elucidated, supporting evidence of oncogenic function may be inferred from other malignancies. TrkB has been implicated in the pathogenesis of neuroblastoma where expression is correlated with poor outcome and a chemo-resistant phenotype [37,38], increased mortality in Wilm's tumor [39], lymph node metastasis and advanced stage in non-small cell lung cancer [40], shorter survival in ovarian cancer [16][15] and distant metastases and poor prognosis in gastric cancer [41]. TrkB has also been found to regulate migration, invasion and epithelial-mesenchymal transition in head and neck squamous cell carcinoma [23]. Signalling via the BDNF/TrkB pathway stimulates pro-survival signals, resistance to anoikis and altered cellular aggregation, all features of cancer cells and prerequisites of metastases formation [42]. Emerging roles for NTs in angiogenesis provide further insight into their potential relevance to cancer development and progression [43]. Studies have identified BDNF and TrkB as key mediators of vascular development [44]. BDNF is an endothelial survival factor, deficiency of which results in reduced endothelial cell-cell contacts and apoptosis [45]. BDNF has been implicated as a novel angiogenic protein in multiple myeloma [14,46]. In support of this, BDNF activation of TrkB has been found to induce vascular endothelial growth factor (VEGF) expression via hypoxia inducible factor-1-alpha (HIF-1-α) in neuroblastoma cells [47]. It has also been suggested that increased expression of TrkB/BDNF may indicate increased neo-neurogenesis, where tumors initiate their own innervation by releasing neurotrophic factors [48]. This may further support tumour progression by the release of neurotransmitters which enhance metastasis. Indeed, the presence of nerve cell markers in tumoral tissue has been found to be a prognostic marker in several human malignancies [49]. In addition to utility as a prognostic marker, therapeutic manipulation of the TrkB signal transduction pathway appears to be an increasingly important target in cancer biology which merits further exploration [11,50]. Despite the inferences drawn, the mechanisms through which BDNF exerts its oncogenic activity have yet to be adequately determined and this will undoubtedly be necessary to optimise any potential therapeutic applications.

Limitations of the present study include the use of background parenchyma from BC patients to provide 'normal tissue' for comparison is also contentious. Ideally, such material should be derived from patients without BC in order to avoid any 'field change' which may exist within cancer bearing tissues. Although the sample size and follow-up period were substantial, it is possible that a larger cohort, particularly with regard to subgroup analysis, may have influenced several results which approached, but failed to reach, statistical significance. In addition to the measurement of mRNA transcript levels and qualitative immuno-histochemistry, quantitative analysis of protein expression should be undertaken to ensure concordance. Correlation with associated molecules, in particular TrkB and P75^NTR^, and other markers of invasiveness and metastatic competence would also be of value.

## Conclusions

BDNF expression was found to be significantly higher in BC specimens compared to normal tissues. Higher transcript levels were significantly associated with unfavourable pathological parameters including nodal positivity and increasing NPI; and adverse clinical outcomes including LR, death from BC, poor prognosis, reduced DFS and OS. In addition to the prognostic utility of BDNF, further mechanistic studies are warranted to explore the potential for therapeutic manipulation in human BC.

## Competing interests

The authors declare that they have no competing interests.

## Authors' contributions

NP: Literature Review, Data Interpretation, Manuscript Writing & Editing. WJ: Study Design/Concept, Laboratory Methodology, Data Acquisition/Analysis. KM: Principal Investigator, Study Design/Concept, Patient Recruitment, Data Analysis. All authors read and approved the final manuscript.
